# Lifetime posttraumatic stress disorder as a predictor of mortality: a systematic review and meta-analysis

**DOI:** 10.1186/s12888-023-04716-w

**Published:** 2023-04-10

**Authors:** Dinuli Nilaweera, Aung Zaw Zaw Phyo, Achamyeleh Birhanu Teshale, Htet Lin Htun, Jo Wrigglesworth, Caroline Gurvich, Rosanne Freak-Poli, Joanne Ryan

**Affiliations:** 1 School of Public Health and Preventive Medicine, Monash University, Level 5, Melbourne, VIC 3004 Australia; 2 Department of Psychiatry, Central Clinical School, Alfred Hospital and Monash University, Melbourne, VIC 2004 Australia

**Keywords:** PTSD, Posttraumatic stress disorder, Mortality, Death

## Abstract

**Background:**

Posttraumatic Stress Disorder (PTSD) could potentially increase the risk of mortality, and there is a need for a meta-analysis to quantify this association. This study aims to determine the extent to which PTSD is a predictor of mortality.

**Methods:**

EMBASE, MEDLINE, and PsycINFO were searched systematically on 12th February 2020, with updated searches conducted in July 2021, and December 2022 (PROSPERO CRD42019142971). Studies involving community-dwelling participants with a diagnosis of PTSD or PTSD symptoms, and a comparator group of individuals without PTSD, and which assessed mortality risk, were included. A random-effects meta-analysis was conducted on studies reporting Odds Ratio (OR), Hazard Ratio (HR), and Risk Ratio (RR), and subgroup analysis was also performed by age, sex, type of trauma experienced, PTSD diagnosis, and cause of death.

**Results:**

A total of 30 eligible studies of mostly good methodological quality were identified, with a total of more than 2.1 million participants with PTSD. The majority of studies involved male-dominated, veteran populations. PTSD was associated with a 47% (95% CI: 1.06–2.04) greater risk of mortality across six studies that reported OR/RR, and a 32% increased risk across 18 studies which reported time to death (HR: 1.32, 95% CI: 1.10–1.59). There was very high study heterogeneity (I^2^ > 94%) and this was not explained by the prespecified subgroup analysis.

**Conclusion:**

PTSD is associated with increased mortality risk, however further research is required amongst civilians, involving women, and in individuals from underdeveloped countries.

**Supplementary Information:**

The online version contains supplementary material available at 10.1186/s12888-023-04716-w.

## Introduction

Posttraumatic Stress Disorder (PTSD) is a chronic psychological disorder that can arise after exposure to a major traumatic event, such as during war, natural disasters, and serious assault [1, 2]. It is predicted that approximately 70% of adults worldwide have encountered at least one potentially traumatic event throughout their lifetime [3]. Previous estimates have reported that 5.6% of trauma-exposed individuals will develop PTSD during their lifetime [4], which can be diagnosed when individuals clinically present with symptoms including, but not limited to, flashbacks and recurring dreams of the event, avoidance behaviours, and negative alterations to cognition and arousal [5, 6]. The global prevalence of PTSD across 24 countries has been estimated to be 3.9% [4]. However, this rate is reportedly even higher in certain groups of individuals [7]. For example, PTSD is up to four times more prevalent in US combat veterans than in US civilians [8].

The pathophysiology of PTSD consists of neurochemical and neuroendocrine dysfunction [9, 10], including altered functioning of the hypothalamic-pituitary-adrenal (HPA) axis stress response [11–13].Dysregulation of the stress system may lead to deleterious consequences across a range of body systems, and contribute to the risk of comorbidities, such as cardiovascular, metabolic, autoimmune and inflammatory diseases [14–19].

To date, only one pooled analysis published in 2015 of 10 cohort studies has investigated the association between PTSD and the risk of all-cause mortality [20], and reported a 29% increased risk of mortality in individuals with PTSD, versus without PTSD. However, that review was not intended to be a formal meta-analysis, and may not have included all relevant studies.

Several studies have also indicated earlier cause-specific mortality in individuals with PTSD, including infection, cancer and cardiovascular-related death [21–23]. Increasing evidence suggests a relationship between PTSD and cardiovascular disease, with a meta-analysis of 402,274 participants published in 2013 revealing that PTSD was associated with a 55% increased risk of incident coronary heart disease or cardiac mortality [24].

Due to the number of more recent studies published in this field, there is a need for a more updated review. Thus, the primary aim of this systematic review and meta-analysis is to determine the extent to which PTSD predicts the risk of death (all-causes) at a given time, or the time to death, hereafter simply referred to as mortality risk. Sub-group analysis examined this association according to age, sex, type of trauma experienced (civilian or military environment), diagnosis or probable PTSD, type of PTSD symptoms experienced, and cause of death.

## Methods

The systematic review was conducted in accordance with the Preferred Reporting Items for Systematic Reviews and Meta-Analyses (PRISMA) Statement [25], and the ‘PRISMA checklist for systematic reviews’ has been included in Supplementary Appendix 1. The protocol was registered with the International Prospective Register of Systematic Reviews (PROSPERO) (registration number CRD42019142971).

### Inclusion/exclusion criteria

#### Types of studies

Studies were included if they were peer reviewed, original cohort or case-control studies. Reviews, case reports and series, and articles written in a language other than English were excluded.

#### Types of participants and exposures

Studies were included if they involved individuals with a PTSD diagnosis and a comparator group of individuals without a diagnosis of PTSD or PTSD symptoms. We also included participants with a probable diagnosis of PTSD, or PTSD symptoms, as measured by self-administered questionnaires. Subgroup analysis was later conducted to account for the differences between individuals with a formal and probable diagnosis of PTSD (see Sect. 2.5). Amendments to the protocol were made to exclude entire clinical samples with PTSD that met the criteria for another psychiatric diagnosis (e.g. a sample of patients with depression who also displayed PTSD symptoms) or another medical diagnosis (e.g. all PTSD patients who were undergoing coronary angiography). These targeted patient samples were excluded in order to limit the confounding influence of specific medical conditions, which could limit the external generalisability of the findings.

#### Types of outcome measures

The primary outcome of interest was mortality (all-cause as well as specific causes). Where stated, studies investigating only deaths due to suicide were excluded due to the already well-established link between PTSD and suicidal behaviour [26]. In addition, the neurobiological mechanisms underpinning PTSD and suicidal behaviour are likely to differ with that of somatic conditions. The protocol was amended to limit confounding and inflation of the pooled effect estimates because the association between PTSD and suicide is already well recognised [27]. However, studies that reported external causes of death, without distinguishing deaths due to unintentional overdoses, motor vehicle or other accidents, or suicide, were included. Deaths could be self-reported (family member, next-of-kin), from death certificates, medical records, or databases. Secondary outcomes included the cause of death. There were no restrictions regarding the length of follow-up to track mortality.

### Search methods

A systematic search of EMBASE (1947-present), MEDLINE (1946-present), and PsycINFO (1806-present) was conducted through Ovid software. Relevant studies were identified using a combination of subject headings and keywords, which were adapted using truncations and Boolean operators (Supplementary Appendix 2 A-2 C). The population was identified using the following search terms: [posttraumatic stress disorder; (posttraumatic or post traumatic) adj stress; PTSD]. The outcome was identified using the following search terms: [mortality; all-cause mortality; cardiovascular mortality; hospital mortality; mortality rate; mortality rate; premature mortality; accidental death; cause of death; heart death; sudden death; mortalit*; death*]. Searches were conducted on February 12th, 2020 following consultation with a Senior Librarian. An updated search was conducted on the 17th July, 2021, and 9th December, 2022.

### Data collection

Following the initial search, duplicate research articles were removed by one reviewer (DN). The articles were exported to Covidence systematic review software (www.covidence.org) for subsequent reference management. Four reviewers (DN, AP, AT, HH) independently assessed the articles by title and abstract, to determine eligibility for full-text review. Final inclusion for data extraction were determined by four reviewers (DN, AP, AT, HH), with a third reviewer (JR) involved in cases of conflicting verdicts.

One reviewer (DN) extracted the following information from the final set of included articles. Data were first extracted on 27/04/2020 using a standardised data extraction form:

1. Study details (author, year, country, study design, database, baseline, and follow-up years).
2. Participant characteristics (sample size, eligibility crietria, mean age, percentage of women participants).
3. PTSD assessment.
4. Mortality risk estimate (Odds Ratio (OR), Relative Risk (RR), Hazard Ratio (HR), or Survival Ratio (SR)), or descriptive statistics if the risk estimate was not available; and the cause of death.
5. Factors adjusted for in the analysis.

Two reviewers (AP and JW) independently verified extracted data.

### Quality assessment

The quality and risk of bias of the included articles were independently evaluated by four reviewers (DN,JW, AT, HH) using The Joanna Briggs Institute Critical Appraisal Checklist [28] for the relevant study design. Any discrepancies in quality assessment were resolved through discussion, and if consensus could not be reached, by a third reviewer (JR). Ten components of study quality were assessed, including whether: the PTSD and comparator groups were from the same population, PTSD was assessed in both groups, PTSD was measured in a valid and reliable way, confounding factors (age, sex, depression) were identified, strategies to deal with confounding factors were stated, participants were not deceased at the time of diagnosis, mortality was measured in a valid and reliable way, follow-up time was at least one year, follow-up time was complete for at least 80% of participants, and appropriate statistical analysis was used. Articles were scored out of ten, with a score of one to four indicating poor quality, five to seven indicating fair quality, and eight to ten indicating good quality.

The Grading of Recommendations Assessment, Development and Evaluation (GRADE) framework was used to evaluate the quality of evidence in which the following were assessed: risk of bias, inconsistency (based on heterogeneity), indirectness (population differences), imprecision (summary estimate CIs), publication bias (funnel plots and Egger’s test), and pooled effect sizes (OR/RR and HR) [29].

### Data syntheses and meta-analyses

Meta-analyses were conducted with the final included studies using Stata statistical software, version 16.0 (StataCorpLP, College Station, TX, USA). The risk estimates of interest were Odds Ratio (OR) and Risk Ratio (RR), in addition to Hazard Ratio (HR) which provided information regarding time to death. A random-effects approach was used to report the overall mortality risk estimates (OR/RR, HR, 95% CI), to be displayed as forest plots. The risk estimates of OR and RR were combined in the one meta-analysis, as OR can be used to approximate RR when the outcomes are relatively rare [30]. For studies in which more than one estimate was provided, we endeavoured to extract the effect size which would be the most generalisable (e.g. using results from all-cause mortality rather than cause-specific death), adjusted for the covariates of interest (age, sex, depression), and from the largest sample provided.

Heterogeneity was assessed using I² statistics, with the results of this analysis interpreted according to the Cochrane guidelines (0–40% = might not be important; 30–60% = may represent moderate heterogeneity; 50–90% = may represent substantial heterogeneity; and 75–100% = considerable heterogeneity) [31]. Statistical significance was set at P < 0.05. Publication biases were assessed using funnel plots and Egger’s test. Additional subgroup analyses were conducted to investigate factors that may account for heterogeneity in the association between PTSD and mortality. This included according to age, sex, whether participants were military veterans or civilians, follow-up years, whether PTSD diagnoses or a probable diagnosis (based on cut-points from continuous symptom severity scores) was assessed, type of PTSD symptom experienced, cause of death, and risk of bias assessment.

## Results

### Search results

**Fig. 1:**
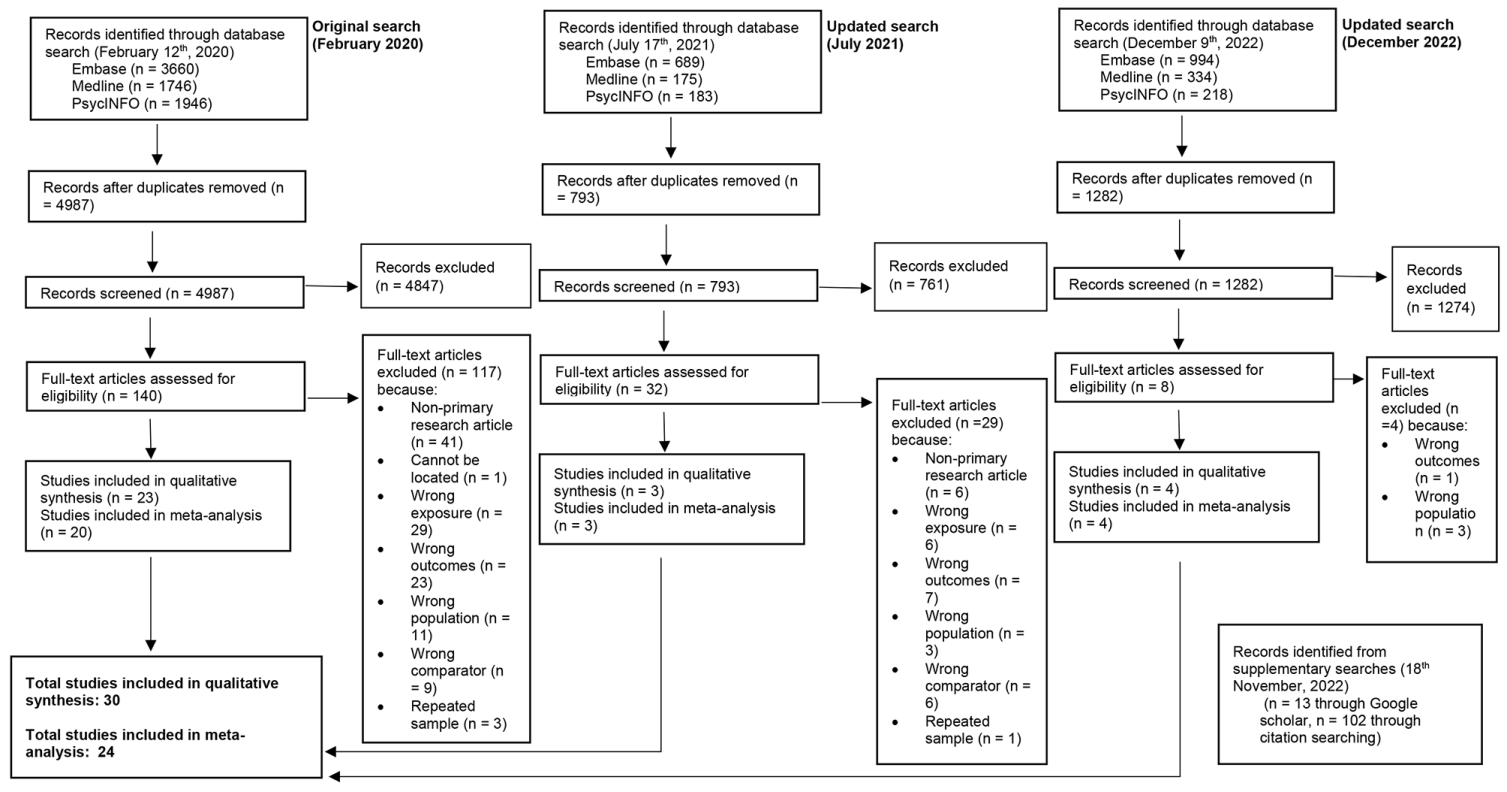
PRISMA flow diagram

The search of EMBASE, MEDLINE and PsycINFO yielded 9,945 articles (Fig. 1). Duplicates were removed (n = 2,883), resulting in 7,062 papers included for subsequent screening. Upon screening by title and abstract, a further 6,882 articles were excluded, as these studies either did not investigate human populations, were not in English, or were nonprimary research articles. One hundred and eighty articles underwent a full-text review. Of these articles, 146 papers were excluded as they did not meet the eligibility criteria. A further four studies were excluded as they utilised the same participants, or a subsample [32–35]. To avoid repeating participants in the meta-analysis, only one study that utilised the same participants was selected [36], and this was based on having a completed sample, and a variety of outcomes (all-cause, external, cardiovascular (CVD) and cancer-related deaths). An additional search of Google Scholar revealed 13 articles, and citation searching yielded 102 articles, however none of these studies proceeded to full-text screening. A total of 30 articles were thus included in this systematic review, with 24 articles included in the primary meta-analysis.

### Study and participant characteristics

The characteristics of the 30 included studies [22, 23, 36–63], encompassing at least 2.1 million participants with a confirmed or probable diagnosis of PTSD are shown in Table 1. All studies were cohort studies, except for one case-control [56]. The period of follow-up ranged from one to 70 years, and the majority of studies (n = 22) had a follow-up of ≤ 10 years. Nineteen studies were conducted in the US, seven in Europe, two in the Middle East, and two in East-Asia. The majority of the included studies focused specifically on veteran military populations, and only 12 studies analysed civilian populations. Of the civilian studies, two involved participants exposed to war trauma [38, 49], two terrorist attacks [41, 51], one a natural disaster [44], and seven included participants exposed to any type of trauma [22, 23, 45, 58–61].

**Table 1:** Characteristics of 30 included studies

Authors and year	Setting, Study or database	Sample size (n) and PTSD %	Age at baseline (mean (SD) or range), female %	Follow-up years	Type of trauma	PTSD assessment	PTSD-mortality association*	Cause of death	Adjustment
Bohnert et al., [37]	US, Prospective, VA NPCD	3,291,891, 6.27%	75.4% between 40–59 yrs, F: 10%	6 yrs	War	Current diagnosis, ICD-9-CM	↑ risk	External (accidental overdose)	Age, sex, other
Boscarino et al., 34	US, Prospective, National Personnel Records Centre	15,288, 6.87%	13-20.4% ≥ 40 yrs: F: 0%	16 yrs	War	Current diagnosis, RTI – PTSD, DIS – III PTSD, and Combat Exposure Scale	↑ risk	All-cause, external, CVD and cancer	Age, sex, other
Bramsen et al., [38]	Netherlands, Prospective, registries of 9 cities	1448, 4.49%	67.3 (2.9) yrs, F: 38%	10 yrs	War (civilians)	Current symptoms, SRIP based on DSM-IV	↑ risk	All-cause	Age, sex, other
Bullman & Kang, [48]	US, Prospective, Agent Orange Registry	16,257, 26.12%	31 yrs, F: 0%	4.2–5.2 yrs	War	Current diagnosis, DSM-III and DSM-III-R	↑ risk	All-cause, cancer, circulatory & digestive, and external	Age, sex, other
Cho et al., [39]	US, Prospective, VHA	80s subsample: 665,249 (total n = 721,588), 1.7%	84 (3.3) yrs, F: 2%	5 yrs	War	Current diagnosis ICD-9-CM	↓ risk	All-cause	Unclear
Chwastiak et al., [57]	US, Prospective, VA administrative records	559,985, 6.2%	64.1 (12.9) yrs F: 4.1%	9 yrs	War	Current diagnosis, ICD-9-CM	Null Subsample: n = 483,091	All-cause	Age, sex, psychiatric disorders, other
Clark et al., [62]	US, Prospective, VA NPCD, CMS, SPAN & MDR	951,018, 50%	62.9 (8.30)-63.6 (7.4) yrs, F: 5.6–6.3%	5.8 yrs	War	Current diagnosis, ICD-9	↑ risk	External (unintentional drug overdose)	Age, sex, psychiatric disorders, other
Flood et al., [40]	US, Prospective, VA PTSD clinic & VES databases	5248, 22.41%	37.84–50.46 yrs, F: 0%	12–15 yrs	War	Current diagnosis, DSM-III-R and DIS-III	↑ risk	All-cause	Age, sex, other
Giesinger et al., [41]	US, Prospective, The WTC Health Registry	63,666, 10.51%	40.4 (10.4), F: 38.9%	13 yrs	Terrorist attack	Current symptoms, PCL-17	↑ risk	All-cause, CVD, external	Age, sex, depression, other
Gradus et al., [59]	Denmark, Prospective, National medical & social registers	512,101, 0.74%	47.8–54% between 31–55 yrs, F: 60-60.1%	16 yrs	None specified	Current diagnosis, ICD-10	↑ risk	All-cause	Depression, other
Kilbourne et al., [55]	US, Prospective, VHA	Total unknown, PTSD: 281,545	Age at death: 65 (12.4) yrs (all-cause), 66.4 (12.3) (CVD), F % unknown	1 yr	War	Unknown	↑ risk YPLLs: All-cause 18.4 ± 8.4 (18.1–18.7), CVD: 17.4 ± 8.1 (6.9–17.9)	All-cause and CVD	Age, sex
Kim et al., [23]	Korea, Prospective, Korean National Health Insurance Database	214,996, 25%	49.86 (11.30)-50.31 (11.54) yrs, F: 64.6%	6–7 yrs	None specified	Current diagnosis, ICD-10	↑ risk	CVD	Age, sex, depression, other
Kimbrell et al., [42]	US, Prospective, VISN 16 data warehouse	Without purple heart subsample: 8603 (total n = 10,255), 41.76%	73.7–73.8 yrs, F: 0.8–1.1%	2 yrs	War	Current diagnosis, ICD-9-CM	↑ risk	All-cause	Age, sex, other
Kinder et al., [43]	US, Prospective, ACQUIP data repository	Without depression subsample: 25,077 (total n = 35,715), 13.39%	58 (13) – 64 (12) yrs, F: 0.5–3.8%	2 yrs	War	Current diagnosis, ICD-9-CM	Null	All-cause	Age, sex, depression, other
Lewandowski-Romps et al., [56]	US, Case-control, Army and Department of Defense administrative records	Cases: 1080, controls: 30,939,614, PTSD unknown	Age unknown, F: 5.6–13.8%	5 yrs	War	Current diagnosis, ICD-9-CM	↑ risk Never deployed OR: 2.8 (1.3–6.1)	External (accidental)	Age, sex, depression, other
Li et al., [44]	Japan, Prospective, The Japan Gerontological Evaluation Study	2,965, 25.2%	73.4 (6.2) yrs, F: 54.7%	3.3 yrs	Natural disaster	Current symptoms, SQD-P	Null	All-cause	Age, sex, depression, other
Meier et al., [60]	Denmark, Prospective, population registries	3,270,650, PTSD unknown	Age unknown, F % unknown	9.7 yrs	None specified	Lifetime diagnosis, ICD-10	↑ risk	All-cause, external, natural causes	Age, sex, depression, other
Mollica et al., [49]	Croatia, Prospective, Bosnian refugees living in camps	528, 26.3%	33.7% between 35–54 yrs, F: 58.4%	3 yrs	War (civilians)	Current diagnosis, DSM-IV and HTQ	Null	All-cause	None
Roberts et al., [45]	US, Prospective, Nurses’ Health Study II	Without depression + 4–5 PTSD symptoms subsample: 13,249 (total n = 51,602), 32.90%	53.2 (4.7)-53.3 (4.6), F: 100%	9 yrs	None specified	Current symptoms, Short Screening Scale for DSM-IV PTSD	Null	All-cause	Age, sex, depression, other
Schlenger et al., [46]	US, Prospective, National Vietnam Veterans Study	Theatre veteran subsample: 1632 (total n = 2348), 10.66%	41.5 yrs, F: 26.5%	24 yrs	War	Current symptoms, Survey assessment, DSM-III (clinical subsample)	↑ risk	All-cause	Age
Solomon et al., [54]	Lebanon, Prospective, war veterans	680, PTSD unknown	29.4 (9.2) − 32.7 (9.3) yrs, F: 0%	33 yrs	War	Current symptoms, PTSD Inventory based on DSM-III	Null	All-cause	Sex, depression, other
Song et al., [21]	Sweden, Prospective, The National Patient Register	Population based cohort: 1,460,731, (total n = 1,881,793), 0.8%	37.2 (14.2) yrs, F: 61.7%	8 yrs	None specified	Current diagnosis, ICD-9 or ICD-10	↑ risk	Infection	Age, sex, psychiatric disorders, other
Szymanski et al., [63]	US, Retrospective, VHA	8,812,373, 9.8%	55–74 yrs: 42.19%, F: 11.7%	1 yr	War	Current diagnosis, ICD-9 or ICD-10	↑ risk	All-cause	Age, sex, other
Tian et al., [22]	Sweden, Prospective, The National Patient Register	Population based cohort: 1,910,873 (total n = 2,409,931), 0.8%	37.3 (14.6) yrs, F: 62.2%	9–10 yrs	None specified	Current diagnosis, ICD-9 or ICD-10	↑ risk	All-cause	Age, sex, psychiatric disorders, other
Tian et al., [22]	Sweden, Prospective, The National Patient Register	Population based cohort: 1,644,414 (total n = 2,080,339), 0.8%	36.6 (14.2)- 36.2 (14), F: 62%	7–8 yrs	None specified	Current diagnosis, ICD-9 or ICD-10	↑ risk	Cancer	Age, sex, other
Trivedi et al., [50]	US, Prospective, VHA’s corporate data warehouse	4,461,208, 9.3%	55.9 (14.2) yrs, F: 7.9%	1 yr	War	Current diagnosis, ICD-9,	↓ risk	All-cause	Age, sex, other
Vaillant et al., [53]	US, Prospective, Harvard University	244, PTSD unknown	19 yrs, F: 0%	70 yrs	War	Current symptoms, Interview & symptoms based on DSM-III,	↑ risk. F(2, 241) = 4.0, P = 0.019	All-cause	Sex, other
Welch et al., [51]	US, Prospective, WTC health registry	1193, 24.5%	48.8% between 45–64 yrs, F: 45.2%	8 yrs	Terrorist attack	Current symptoms, PCL-17	↑ risk	External (alcohol & drug related)	Sex, other
Wolf et al., [52]	US, Prospective, VHA trauma-exposed veterans & partners	339, 69%	52.58 (10.65) yrs, F: 13.0%	6.5 yrs	War	Lifetime symptoms, DSM-IV	Null, subsample: n = 241	All-cause	Age, sex, other
Zohar et al., [47]	Israel, Prospective paired-sample, MOD	4914, 50%	~ 47 yrs, F: 0%	> 9 yrs	War	Current diagnosis, DSM-IV	Null, subsample: n = 4889	All-cause	Age, sex, other

**ACQUIP** = Ambulatory Care Quality Improvement Project, **BMI** = Body Mass Index, **CMS =** Centres for Medicare & Medicaid Services, **CVD** = Cardiovascular Disease, **DIS – III** = Diagnostic Interview Schedule - Version III adapted from the DSM-III, **D-PTSD** = PTSD scale corresponding to the DSM-III, **DSM-III** = The Diagnostic and Statistical Manual of Mental Disorders, third edition, **HTQ** = Harvard Trauma Questionnaire, **ICD-9** = International Classification of Diseases Ninth Revision, **ICD-9-CM** = International Classification of Diseases Ninth Revision Clinical Modification, **IDF** = Israeli Defense Forces, **MDR** = Mortality Data Repository, **MOD** = Ministry of Defense, **NPCD** = National Patient Care Database, **PCL-17** = PTSD Checklist-Stressor Specific Version 17, **RTI** = The Research Triangle Institute, **SPAN** = Suicide Prevention Applications Network, **TBI** = Traumatic Brain Injury, **VA** = Veterans Affairs, **VHA** = Veterans Health Administration

**Fig. 2:**
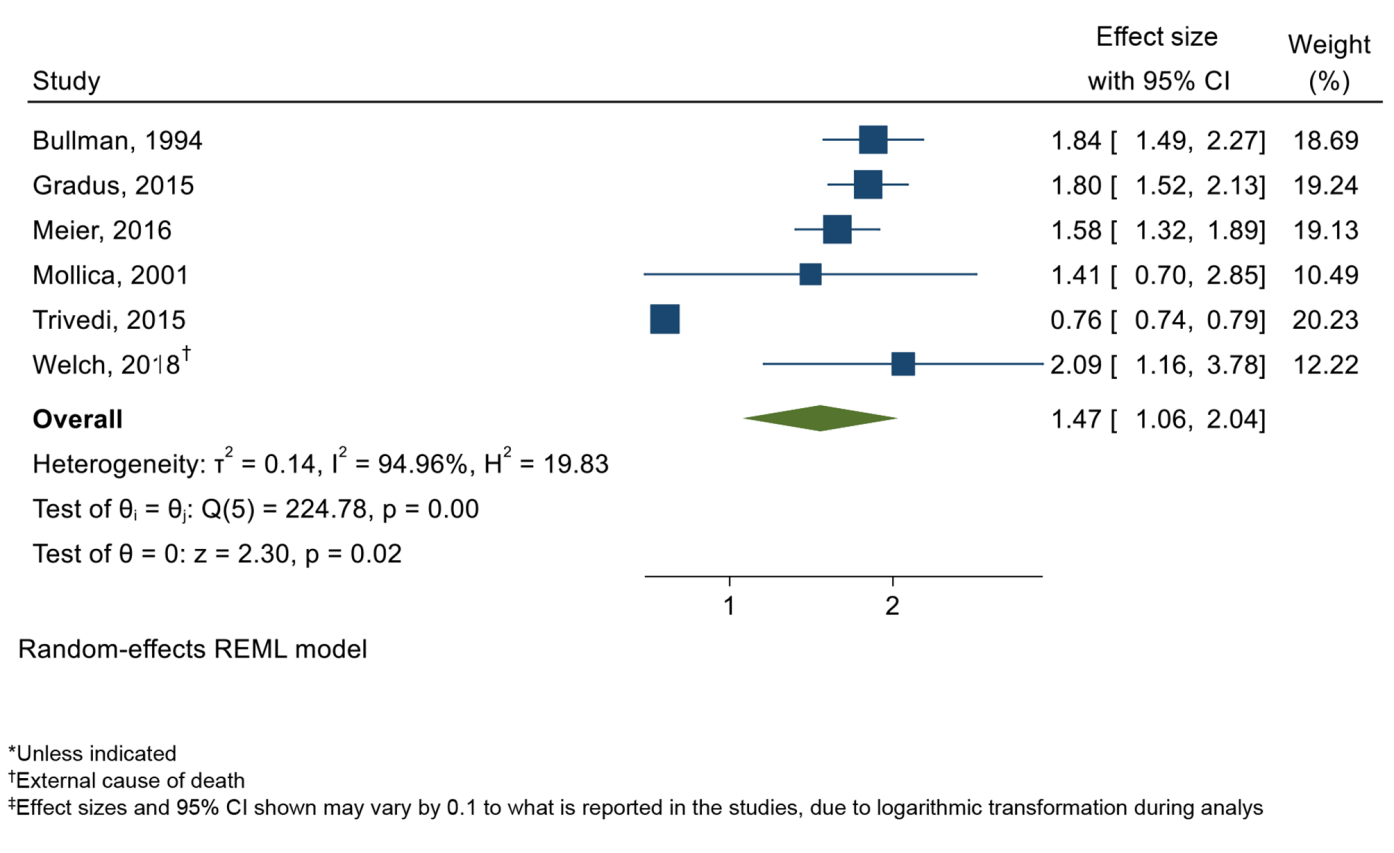
Forest plot displaying pooled analysis of PTSD and all-cause mortality*, reporting Odds Ratio (OR) or Risk Ratio (RR)^‡^ (n = 6)

**Fig. 3:**
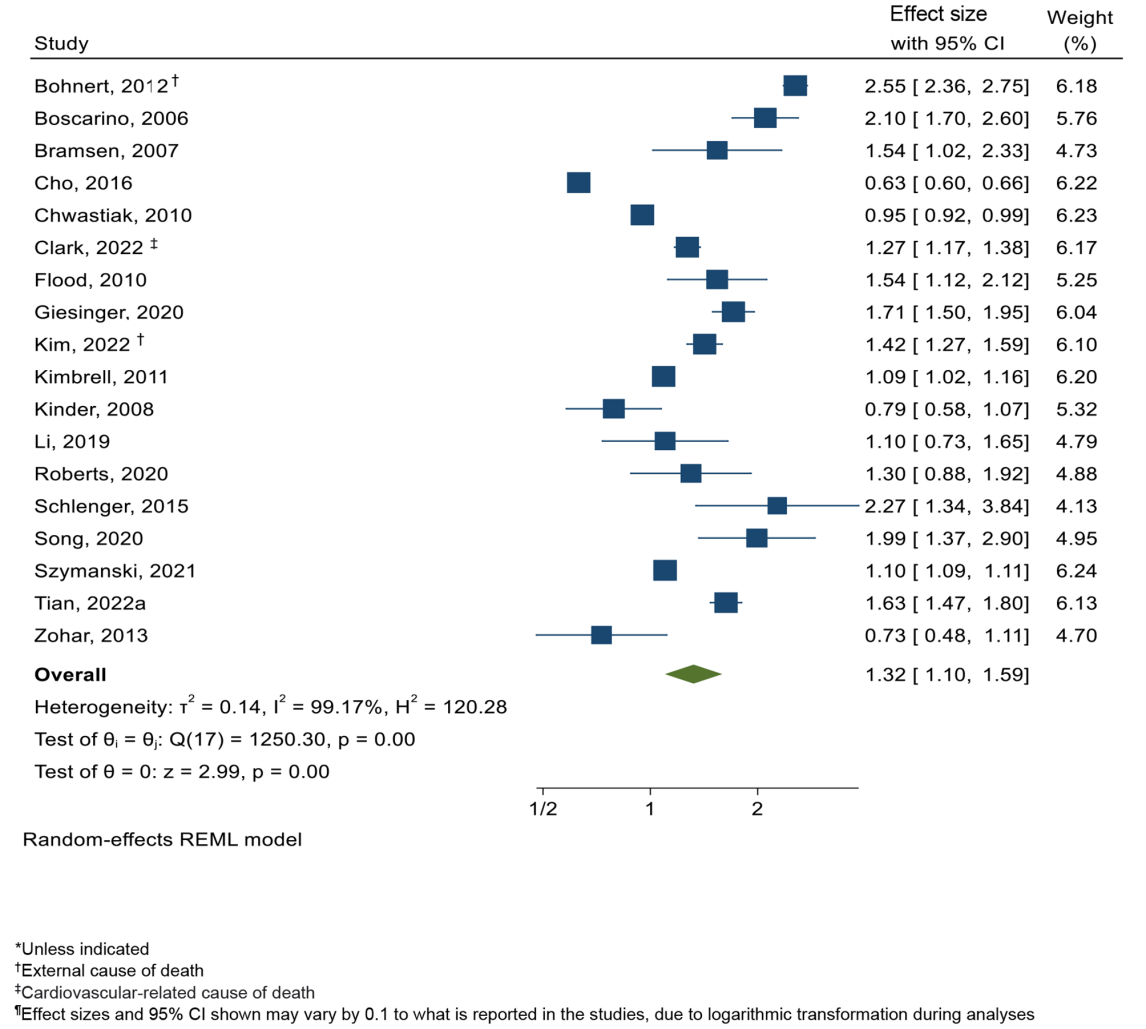
Forest plot displaying pooled analysis of PTSD and all-cause mortality*, reporting Hazard Ratio (HR) ^¶^ (n = 18)

*Effect sizes are shown in Figures 2-3. Those shown here could not be included in the meta-analysis.

The majority of samples were male-dominated, with six studies involving only men, and only one study with women exclusively [45]. Amongst the studies that included both sexes, the percentage of women ranged from 0.5 to 64.6%. One study did not specify the proportion of each sex [60], and one study did not specify if both sexes were included [55].

The percentage of participants with PTSD varied between 0.74% and 69%. PTSD was most frequently diagnosed using the International Classification of Diseases, Ninth Revision, Clinical Modification (ICD-9-CM) [64]. A total of nine studies [38, 41, 44–46, 51–54] investigated a probable diagnosis of PTSD.

All but seven studies [22, 23, 37, 51, 56, 58, 62] investigated all-cause mortality. While we have excluded studies that focused specifically on suicide, four studies [36, 48, 60, 62] reported findings for all external causes of death (which included suicide, homicide and accidental deaths). Four studies analysed CVD-related deaths [23, 36, 41, 55], three studies reported deaths by cancer [22, 36, 48], and one study investigated infection-related deaths [58].

### Risk of bias assessment

The risk of bias scores assessing methodological quality of the included studies is shown in Supplementary Appendix 3 A-3B. Fifteen articles were rated as good quality (score 8–10), 14 as fair [4–7], and one only as poor quality (≤ 3) [55]. The most common sources of potential bias were participants without PTSD not having undergone PTSD assessment (due to many studies obtaining diagnoses via medical records rather than performing an assessment in the entire sample), failure to identify and deal with potential confounding factors (in particular depression), and lack of information regarding whether follow-up was complete. Quality of evidence was assessed by GRADE criteria. Of the five categories, the only serious issue was inconsistency, due to high unexplained heterogeneity (Supplementary Appendix 4). However, given all studies were observational, thus initially classified as ‘low’ overall anyway, this rating was further downgraded to ‘very low’ [65].

### Meta-analyses

Twenty-four cohort studies provided the estimates used in the meta-analyses. All-cause mortality was used as the outcome in all but three studies that only provided estimates for external causes of death [37, 51, 62], and one study which only provided an estimate for cardiovascular-related cause of death [23]. One study [39] provided a survival ratio (SR = 1.37 (1.34–1.40), that was converted into a risk estimate (estimate = 0.63 (0.6–0.66)). Two studies that showed a positive association between PTSD and mortality were excluded from meta-analyses as they did not provide any comparable statistics [53, 55] (Table 1). The one case-control study [56] was too heterogenous (in terms of study design and temporal sequence) to include in the meta-analyses. However, that study demonstrated a 2.8-fold increased risk of mortality amongst soldiers with PTSD. Amongst the studies that measured symptoms or severity of PTSD, two (which presented null findings) were based on a dimensional metric [52, 54] (rather than a 0 vs. 1 determination to indicate probable PTSD diagnosis), and were excluded from analyses. An additional article was also excluded from the main meta-analyses [22] as it utilised the same participants as another study [61], but was later included in the subgroup analyses investigating causes of death.

Meta-analyses were conducted to investigate the overall association between PTSD and mortality. Across the six studies that provided estimates for odds and risk ratios, there was a 47% (95% CI: 1.06–2.04, P = 0.02) greater risk of mortality (Fig. 2). However, substantial heterogeneity was also observed (I^2^ = 94.96%). There was also some evidence of publication bias, as observed by asymmetry in the funnel plots (Supplementary Appendix 5 A), however this was not significant after conducting Egger’s test (P = 0.30).

Similarly, a positive association was observed between PTSD and time-to-death across the 18 studies reporting hazard ratios (HR: 1.32, 95% CI: 1.10–1.59, P = 0.00). (Fig. 3). Very high heterogeneity was observed (I^2^ = 99.17%), with no strong evidence of publication bias (Supplementary Appendix 5B) (Egger’s test, P = 0.53).

### Subgroup analyses

Subgroup analyses were performed to investigate potential sources of heterogeneity and to see whether the association between PTSD and mortality differed according to specific study characteristics.

*Civilian vs. veteran*: Among four studies of civilians [49, 51, 59, 60] PTSD was associated with an 70% greater risk (OR/RR 95% CI: 1.51–1.91, I^2^ = 0.00%, k (number of studies) = 4) (Supplementary Appendix 6 A). Likewise, seven studies investigating time to death showed that risk was 55% greater in civilians (HR 95% CI: 1.41–1.71, I^2^ = 39.82%, k = 7). No significant association was found for the subgroup of studies in veteran populations (k = 11) (Supplementary Appendix 6B).

*Diagnosis vs. probable PTSD*: There was a 55% greater risk (HR 95% CI: 1.27–1.87, I^2^ = 39.68%, k = 5) of mortality in studies that measured probable PTSD, whilst no significant association was found for studies that included a diagnosis of PTSD (k = 13) (Supplementary Appendix 7).

*PTSD symptoms*: Among the studies which included PTSD symptoms, only one article provided an effect size for mortality risk within individual symptom categories (intrusion, avoidance, arousal) [38], thus no subgroup analysis was undertaken.

*Cause of death*: PTSD was associated with an increased risk of external causes of death (HR: 2.03, 95% CI: 1.50–2.74, I^2^ = 94.07%, k = 5) [36, 37, 40, 41, 62], cancer-related deaths (HR 95% CI: 1.14–1.66, I^2^ = 0.00%, k = 2) [22, 36] and CVD-related deaths (HR 95% CI: 1.30–1.81, I^2^ = 32.66%, k = 3) [23, 36, 41] (Supplementary Appendix 8 A). Likewise, similar results were observed in studies that provided OR/RR estimates (Supplementary Appendix 8B).

*Sex*: Given that most studies involved a majority of men (with many only involving men), it was not possible to investigate subgroups defined by sex (Supplementary Appendix 9).

*Follow-up period*: The majority of studies had a follow-up period of ≤ 10 years but there were no clear differences observed in the subgroup analysis (Supplementary Appendix 10). [59].

*Methodological quality of the studies*: Similar trends were observed across all methodological quality subgroups (Supplementary Appendix 11 A and B).

## Discussion 

### Summary of evidence

This systematic review summarises the findings of 30 longitudinal studies examining the association between PTSD and mortality. Studies were of fair-good methodological quality and included at least 2.1 million participants with a PTSD diagnosis or probable PTSD, who were primarily male military veteran populations. Meta-analyses indicated that PTSD increased mortality risk by approximately 47% in six studies reporting OR/RR, and 32% in 18 studies examining time to death, when compared to individuals without PTSD. However, it is important to acknowledge the very high heterogeneity in the interpretation of the meta-analyses, and subgroup analyses did not account for this heterogeneity.

There was a significantly increased risk of mortality with PTSD among civilian populations, but not among the veteran subgroup. Civilian traumas, including natural disasters and sexual assault are generally unexpected, compared to military personnel who are trained and prepared for the trauma-exposure. Some research suggests that a combination of accessibility to treatment and awareness of the disorder within veteran groups promotes greater management of PTSD than in the general population [7]. Of note however, the heterogeneity remained high in the veteran subgroup, but was much lower for the civilians. [66]. When investigating subgroups according to cause of death, the largest effect size was observed for external causes of death. While the focus of this review was on unintentional causes of death, studies investigating external causes of death did not distinguish suicide from other external causes (e.g. homicide). Thus, it is possible that suicides partly account for our finding that PTSD had a stronger association with external causes of death. We also found that PTSD was associated with cancer-related and CVD-related death, with previous studies also describing a relationship between PTSD and incidence of these diseases [67–69]. It has been hypothesised that an overactivity of the sympathetic nervous system in individuals with PTSD, could increase basal heart rate and cause endothelial dysfunction, and subsequently lead to cardiac events. However, it is possible that health problems, including acute cardiovascular events [70] are sufficient to precipitate PTSD in patients, hence it is sometimes difficult to establish whether they lie on the causal pathway with PTSD, or an independent risk factor for mortality.

### Comparison with literature

These findings are largely consistent with a previous pooled analysis [20] that found that participants with PTSD have a 29% greater risk of mortality than those without PTSD. Unlike the present study, that analysis only consisted of 10 papers that excluded hazard ratio information and extensive subgroup analysis. Thus, our findings based on 30 studies conducted using a thorough systematic review, substantially adds to this previous work. In contrast, five independent studies (included in this review) found PTSD to be associated with a reduced mortality risk [39, 43, 47, 50, 57]. These results may be attributed to psychological resilience, as evidence suggesting that experiencing a traumatic event may provide resilience to physical morbidity and mortality, with reduced dementia incidence observed in a France cohort who had undergone lifetime trauma [71]. Thus, it is possible that the populations in studies showing reverse associations may have unique resilience factors, which could also account for the very high heterogeneity observed in our pooled meta-analysis.

Survivorship effects may also explain negative associations between PTSD and mortality, and are supported by our subgroup analyses which did not show excess mortality in studies ≥ 20 years. Specifically, those who survive to older age may be in greater physical and mental health, and be utilising more healthcare services that may be beneficial for longevity. The lack of excess mortality in older-aged adults (60 years and above) by three included studies [39, 50, 57] supports this theory. However, due to the large age ranges included, we were unable to investigate subgroups according to age.

### Potential confounders

While various morbidities may lie on the causal pathway in the association between PTSD and mortality, they may also have a confounding effect independent of shared pathogenesis with PTSD. It has been suggested that people with PTSD are more likely to experience more severe service-connected comorbidities, which may independently be associated with up to a 2.5-fold increased risk of one-year mortality [72].The presence of PTSD may increase patients’ risk of morbidities such as cancer, diabetes, neurodegeneration, gastrointestinal disease, and autoimmune disorders, which may subsequently increase mortality [73]. One explanation may be due to elevated proinflammatory cytokines (e.g., tumour necrosis factor-α and interleukin-6), and reactive oxygen species, which can occur with neuroendocrine changes in PTSD [14, 74], and may be associated with the pathologies of the aforementioned chronic diseases. Nine studies included in this review [22, 43, 45, 50, 57, 58, 61–63] adjusted for physical comorbidities, and four of these studies were not able to indicate excess mortality with PTSD [43, 45, 50, 57]. Mild traumatic brain injury (mTBI) is one possible illness driving the PTSD/mortality association as this is a common occurrence with PTSD [75]. However, despite this, mTBI was adjusted by only two eligible studies [56, 62]. Thus, it is difficult to establish whether it is PTSD, or such other morbidities, that are responsible for increasing mortality risk in our results.

Psychiatric comorbidities are highly prevalent in PTSD affected individuals, with more than 90% of people with PTSD also having another lifetime mental disorder [76]. However, only four studies [57, 58, 61, 62] considered all psychiatric comorbidities in their analyses, and only one of these studies did not demonstrate an increased risk of mortality [57]. Major Depressive Disorder (MDD) or depressive symptoms, which many of the included studies did not adjust for, may explain the strong association observed with external causes of death, as suicidality risk is increased combined with PTSD [77]. Adjusting for psychiatric disorders in a study investigating PTSD as an exposure is a challenge, as other conditions may also share trauma as a common aetiology [78]. However, adjusting for these factors may be unnecessary in the possibility that PTSD may precipitate the formation of other psychiatric disorders, leading to subsequent mortality.

### Generalisability of the findings

A large proportion of participants were US veterans and men, and the current evidence may thus not be widely generalisable to women in the general community. As US veterans have access to specialised healthcare through the Veterans Health Administration [79], our findings may not be applicable to the large majority of the US population who don’t have access to this service or have private insurance. Healthcare utilisation could improve survival and may partially explain the negative association observed in three US veteran studies [39, 43, 50]. In addition to healthcare utilisation, PTSD may be better diagnosed in veterans, due to being an at-risk population. The included studies may have also misrepresented the true number of PTSD patients in the population, due to the challenges in diagnosing PTSD. As PTSD diagnoses were obtained through medical records in the majority of studies, it is possible that there may be undiagnosed participants, which could dilute the true association with mortality. It is likely that the stigma associated with mental illness would be a barrier in diagnosing PTSD, particularly within military environments [80]. Misdiagnoses may have also occurred, as PTSD has overlapping symptoms with other psychiatric disorders [81].

### Strengths

This review was conducted in adherence with the Preferred Reporting Items for Systematic Reviews and Meta-Analyses (PRISMA) statement. Our searches were conducted across three electronic databases, including a grey literature search. Studies were also screened and extracted independently by multiple reviewers. All included studies were longitudinal, were of mostly good methodological quality, and had follow-up periods of at least one year, with only a few studies with follow-up time of less than two years. We included studies with diagnoses and symptoms of PTSD, and the majority of studies also had a valid measure of mortality from nationwide death registries, as opposed to self-report by relatives.

### Limitations

Despite the aforementioned strengths of this review, there was substantial heterogeneity between studies (I^2^ > 94%), which contributed to the ‘very low’ rating in the GRADE assessment. Thus, the unexplained heterogeneity limits the overall conclusions that can be drawn. It is likely that the heterogeneity of our analyses was increased due to our primary outcome being all-cause mortality, which also included studies that investigated any cause of mortality. However, we also performed subgroup analysis where we examined specific cause of death, and variation in length of follow-up may also be a source of heterogeneity in our analyses. We were also unable to perform subgroup analysis based on type of PTSD assessment (e.g. interview or self-report), as many of the included studies did not specify the delivery of this assessment, due to PTSD status being ascertained from chart review. It is likely that structured diagnostic interview assessments, such as the Clinician-Administered PTSD Scale for DSM-5, would provide more reliable and valid determinations of PTSD [82]. Additionally, only some studies adjusted for depressive symptoms and disorders, which may explain the strong associations observed with death by external causes. It is also possible that not all of the included studies were independent, especially studies analysing databases of US veterans, in which the same participant may be included across multiple studies. This is of concern, as overlapping participants could lead to underestimated standard errors and confidence intervals, thus overestimating the precision of our overall estimates [83]. Furthermore, a major source of bias affecting the methodological quality of the studies included not measuring PTSD in the comparator group. Thus, these studies are likely to have a higher percentage of individuals affected by PTSD, thus the provided estimates may be diluted. As we only included three databases in our search strategy, it is possible we may have excluded relevant articles in our analyses. Therefore, the conclusions should be considered within the constraints of these limitations.

### Future directions and conclusion

Our systematic review and meta-analysis is the first comprehensive systematic review and meta-analysis to provide evidence of PTSD increasing the risk of mortality. However, the high unexplained heterogeneity of our findings, should be considered in the interpretation of these results.

The findings of our review also highlight several gaps in the literature which need to be addressed. Prior literature suggests that PTSD disproportionately affects more women than men [7], however only eight included articles included a greater than 50% representation from women [21–23, 44, 45, 49, 59, 61]. While half of these articles have been published in the last two years, suggesting a shift towards equal gender representation in contemporary research, there is still a need for more studies with women. A possible future research question could also be to request female-only data or sex split data from the samples in this review, to ascertain whether a sex difference may be occurring with these results. Furthermore, only 12 included studies analysed civilians, with seven of those articles reporting associations with non-specific traumas [22, 23, 45, 58–61]. While PTSD has historically been referred to as a military disorder, it is increasingly becoming more prevalent among civilians, especially after the COVID-19 pandemic [84]. Thus, further research is required within civilian populations, particularly in countries outside of the US.

Findings from this review highlight the long-term implications of PTSD and may be beneficial in promoting prevention and treatment strategies for this highly debilitating disorder. This review provides preliminary evidence to precipitate future studies, that could investigate whether treating PTSD could reverse premature mortality and aid longevity.

## Electronic supplementary material

Below is the link to the electronic supplementary material.

Supplementary Material 1 The PRISMA checklist for systematic reviews

## Data Availability

The datasets used and/or analysed during the current study available from the corresponding author on reasonable request.

## List of Abbreviations

CI: Confidence Interval
CVD: Cardiovascular Disease
GRADE: Grading of Recommendations Assessment Development and Evaluation
HR: Hazard Ratio
ICD-9-CM: International Classification of Diseases Ninth Revision Clinical Modification
MDD: Major Depressive Disorder
mTBI: Mild Traumatic Brain Injury
OR: Odds Ratio
PRISMA: Preferred Reporting Items for Systematic Reviews and Meta-Analyses
PROSPERO: International Prospective Register of Systematic Reviews
PTSD: Posttraumatic Stress Disorder
RR: Risk Ratio
SR: Survival Ratio
