# Supplementary material for: Lifetime posttraumatic stress disorder as a predictor of mortality: a systematic review and meta-analysis
Source: BMC Psychiatry. 2023 Apr 10;23:229. doi: 10.1186/s12888-023-04716-w (PMC10084620; doi:10.1186/s12888-023-04716-w)
Supplement: Supplementary file 1 — Supplementary Material 1 The PRISMA checklist for systematic reviews [file 12888_2023_4716_MOESM1_ESM.docx]

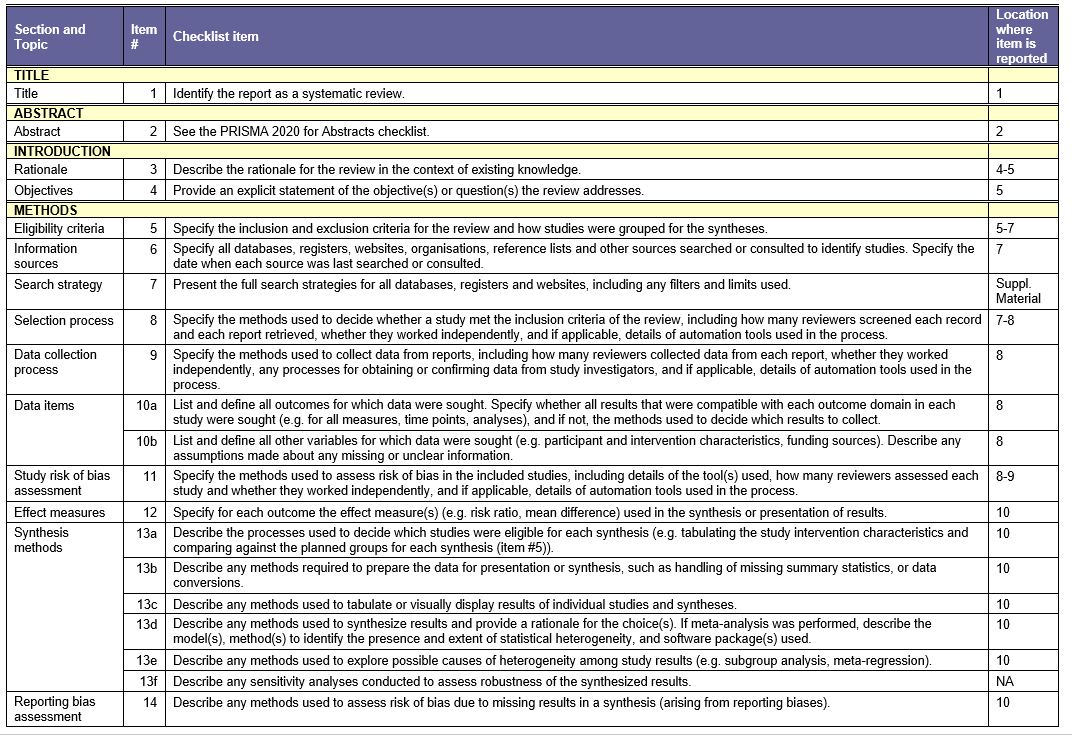
**Supplementary Appendix 1: The PRISMA checklist for systematic reviews**


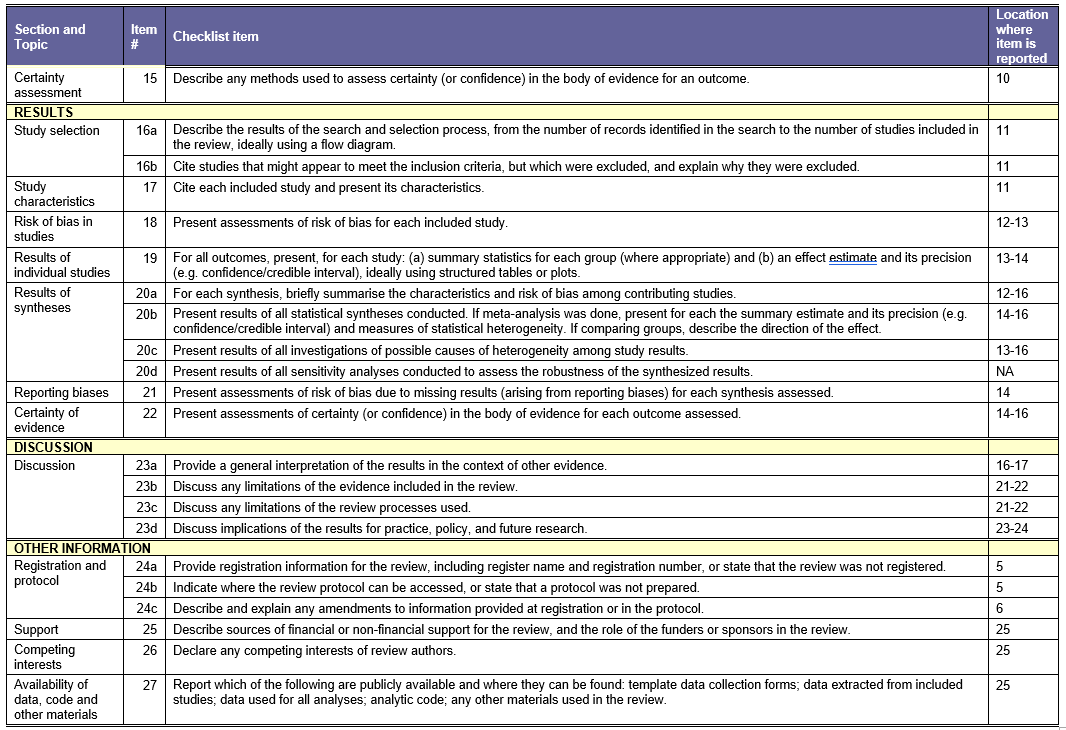


*From:*  Page MJ, McKenzie JE, Bossuyt PM, Boutron I, Hoffmann TC, Mulrow CD, et al. The PRISMA 2020 statement: an updated guideline for reporting systematic reviews. BMJ 2021;372:n71. doi: 10.1136/bmj.n71

**Supplementary Appendix 2A: Search strategy for EMBASE (1947-present)**

| 1. | posttraumatic stress disorder/ |
| --- | --- |
| 2. | ((Posttraumatic or post traumatic) adj stress).mp. |
| 3. | Ptsd.mp. |
| 4. | 1 or 2 or 3 |
| 5. | mortality/ or all cause mortality/ or cardiovascular mortality/ or hospital mortality/ or mortality rate/ or premature mortality/ or accidental death/ or cause of death/ or heart death/ or sudden death/ |
| 6. | Death*.mp. |
| 7. | Mortalit*.mp. |
| 8. | 5 or 6 or 7 |
| 9. | 4 and 8 |

**Supplementary Appendix 2B: Search strategy for MEDLINE (1946-present)**

| 1. | stress disorders, traumatic/ or stress disorders, post-traumatic/ |
| --- | --- |
| 2. | ((posttraumatic or post traumatic) adj stress).mp. |
| 3. | ptsd.mp. |
| 4. | 1 or 2 or 3 |
| 5. | mortality/ or cause of death/ or hospital mortality/ or mortality, premature/ or death, sudden/ |
| 6. | death*.mp. |
| 7. | mortalit*.mp. |
| 8. | 5 or 6 or 7 |
| 9. | 4 and 8 |

**Supplementary Appendix 2C: Search strategy for PsycINFO (1806-present)**

| 1. | exp Posttraumatic Stress Disorder/ |
| --- | --- |
| 2. | ((Posttraumatic or post traumatic) adj stress).mp. |
| 3. | Ptsd.mp. |
| 4. | 1 or 2 or 3 |
| 5. | Death*.mp. |
| 6. | Mortalit*.mp. |
| 7. | 5 or 6 |
| 8. | 4 and 7 |

| **Study** | **Groups from the same population** | **Exposure measured similarly for all** | **Valid measure of exposure** | **Confounding factors identified (age, gender, depression)** | **Strategies to deal with confounding** | **Free of outcome at start of study** | **Valid measure of outcome** | **Sufficient follow-up time** | **Complete follow up** | **Appropriate statistics** | **Total /10** |
| --- | --- | --- | --- | --- | --- | --- | --- | --- | --- | --- | --- |
| Bohnert, 2012 | Yes | No | Yes | No | No | Yes | Yes | Yes | Yes | Yes | 7 |
| Boscarino, 2006 | Yes | Yes | Yes | No | No | Yes | Yes | Yes | Yes | Yes | 8 |
| Bramsen, 2007 | Yes | Yes | Yes | No | No | Yes | Yes | Yes | Yes | Yes | 8 |
| Bullman, 1994 | Yes | No | Yes | No | No | Yes | Yes | Unclear | Yes | Yes | 6 |
| Cho, 2016 | Yes | No | Yes | Unclear | Unclear | Yes | Yes | Yes | Unclear | Yes | 6 |
| Chwastiak, 2010 | Yes | No | Yes | Yes | Yes | Yes | Yes | Yes | Unclear | Yes | 8 |
| Clark, 2022 | Yes | No | Yes | Yes | Yes | Yes | Yes | Yes | Unclear | Yes | 8 |
| Flood, 2010 | No | No | Yes | No | No | Yes | Yes | Yes | Unclear | Yes | 5 |
| Giesinger, 2020 | Yes | Yes | Yes | Yes | Yes | Yes | Yes | Yes | Yes | Yes | 10 |
| Gradus, 2015 | Yes | No | Yes | No | No | Yes | Yes | Yes | Unclear | Yes | 6 |
| Kilbourne, 2009 | Yes | Unclear | Unclear | No | No | Yes | Yes | Unclear | Unclear | No | 3 |
| Kim, 2022 | Yes | No | Yes | Yes | Yes | Yes | Yes | Yes | Yes | Yes | 9 |
| Kimbrell, 2011 | Yes | No | Yes | No | No | Yes | No | Yes | Yes | Yes | 6 |
| Kinder, 2008 | Yes | No | Yes | Yes | Yes | Yes | Yes | Yes | Unclear | Yes | 8 |
| Li, 2019 | Yes | Yes | Yes | Yes | Yes | Yes | Yes | Yes | Yes | Yes | 10 |
| Meier, 2016 | Yes | No | Yes | Yes | Yes | Yes | Yes | Yes | Yes | Yes | 9 |
| Mollica, 2001 | Unclear | Yes | Yes | No | No | Yes | No | Yes | Yes | Yes | 6 |
| Roberts, 2020 | Yes | Yes | Yes | Yes | Yes | Yes | Yes | Yes | Yes | Yes | 10 |
| Schlenger, 2015 | Yes | No | No | No | No | Yes | Yes | Yes | Unclear | Yes | 5 |
| Solomon, 2019 | Yes | Yes | Yes | No | No | Yes | Yes | Yes | Yes | Yes | 8 |
| Song, 2020 | Yes | No | Yes | Yes | Yes | Yes | Yes | Yes | Yes | Yes | 9 |
| Szymanski, 2021 | Yes | No | Yes | No | No | Yes | Yes | Yes | Yes | Yes | 7 |
| Tian, 2022a | Yes | No | Yes | Yes | Yes | Yes | Yes | Yes | Yes | Yes | 9 |
| Tian, 2022b | Yes | No | Yes | Yes | No | Yes | Yes | Yes | Yes | Yes | 8 |
| Trivedi, 2015 | Yes | No | Yes | No | No | Yes | Yes | Yes | Unclear | Yes | 6 |
| Valliant, 2018 | Yes | Yes | No | No | No | Yes | Unclear | Yes | Yes | Yes | 6 |
| Welch, 2018 | Yes | Yes | Yes | No | No | Yes | Yes | Yes | Yes | Yes | 8 |
| Wolf, 2018 | Unclear | Yes | Yes | No | No | Yes | No | Yes | Unclear | Yes | 5 |
| Zohar, 2013 | Yes | Unclear | Yes | No | No | Yes | Yes | Yes | Yes | Yes | 7 |

**Supplementary Appendix 3A**. Critical Appraisal of Cohort Studies Included in the Review, using relevant criteria from the JBI Critical Appraisal Checklist.

**Supplementary Appendix 3B**. Critical Appraisal of Case-Control Studies Included in the Review, using relevant criteria from the JBI Critical Appraisal Checklist.

| **Study** | **Groups were comparable** | **Cases and controls matched appropriately** | **Same criteria to identify cases and controls** | **Valid measure of exposure** | **Exposure measured the same for cases and controls** | **Confounding factors identified (age, gender, depression)** | **Strategies to deal with confounding** | **Valid measure of outcome** | **Sufficient exposure period** | **Appropriate statistics** | **Total/10** |
| --- | --- | --- | --- | --- | --- | --- | --- | --- | --- | --- | --- |
| Lewandowski-Romps, 2018 | Yes | No | Yes | Yes | Yes | Yes | Yes | Yes | Yes | Yes | 9 |

**Supplementary Appendix 4.** Grading of Recommendations, Assessment, Development and Evaluation (GRADE) assessment.

|  |  |  |  |  |  |  |  |
| --- | --- | --- | --- | --- | --- | --- | --- |

| **Odds Ratio/ Risk Ratio** | | | | | | | | |
| --- | --- | --- | --- | --- | --- | --- | --- | --- |
| **No. studies**  **Design** | **Risk of bias** | **Inconsistency** | **Indirectness** | **Imprecision** | **Publication bias** | **Effect** | | **Quality of evidence** |
| 6 studies  Observational | Not serious | Serious^a^ | Not serious | Not serious | Not serious | 1.47 (1.06-2.04) | | Very low |
| **Hazard Ratio** | | | | | | | | |
| **No. studies**  **Design** | **Risk of bias** | **Inconsistency** | **Indirectness** | **Imprecision** | **Publication bias** | **Effect** | **Quality of evidence** | |
| 18 studies  Observational | Not serious | Serious^a^ | Not serious | Not serious | Not serious | 1.32 (1.10-1.59) | | Very low |

^a^The score was downgraded due to substantial heterogeneity between studies which could not be fully explained

**Supplementary Appendix 5A.**  Funnel plot of PTSD and mortality in studies reporting Odds Ratios (OR) and Risk Ratios (RR)

**Supplementary Appendix 5B.**  Funnel plot of PTSD and mortality in studies reporting Hazard Ratios (HR)


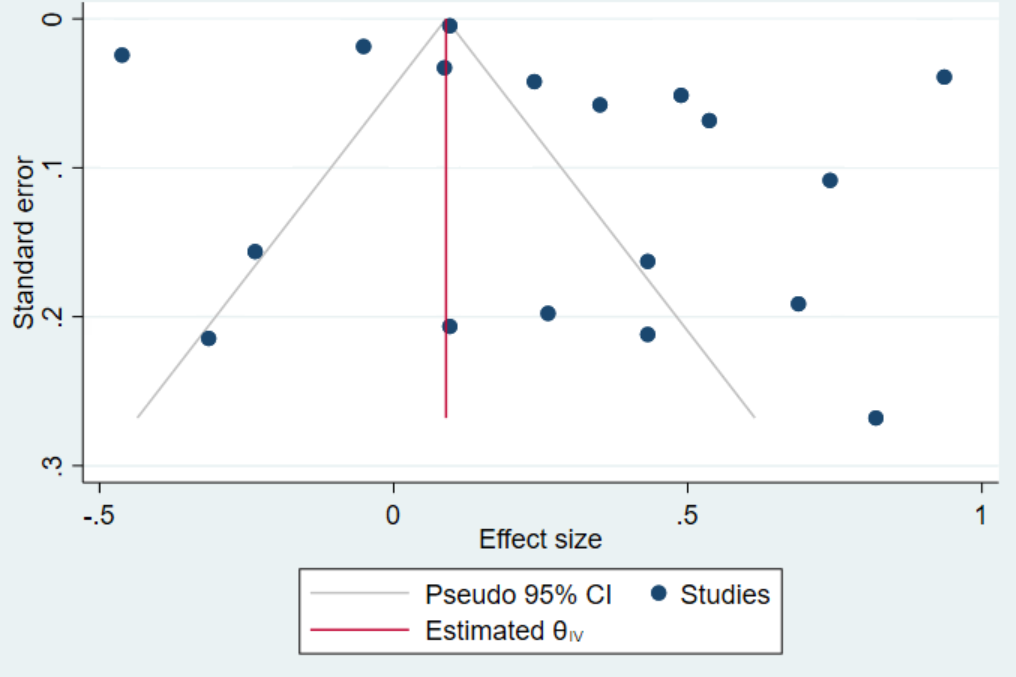


**Supplementary Appendix 6A.** Results from subgroup analyses by veteran status, in studies reporting Odds Ratios (OR) and Risk Ratios (RR)*

**Veterans**

**Civilians**

*Effect sizes shown may vary by 0.1 to what is reported in the studies, due to logarithmic transformation during analyses

**Supplementary Appendix 6B.** Results from subgroup analyses by veteran status, in studies reporting Hazard Ratios (HR)*

**Veterans**

**Civilians**

*Effect sizes shown may vary by 0.1 to what is reported in the studies, due to logarithmic transformation during analyses

**Supplementary Appendix 7.** Results from subgroup analyses by PTSD diagnosis, in studies reporting Hazard Ratios (HR)*

**Diagnosis**

**Probable diagnosis**

*Effect sizes shown may vary by 0.1 to what is reported in the studies, due to logarithmic transformation during analyses

**Supplementary Appendix 8A.** Results from subgroup analyses by cause of death, in studies reporting Hazard Ratios (HR)*

**All-cause**

**External**

**Cancer**

**All-cause**

**External**

**Cancer**

**Cardiovascular**

*Effect sizes shown may vary by 0.1 to what is reported in the studies, due to logarithmic transformation during analyses

**Supplementary Appendix 8B.** Results from subgroup analyses by cause of death, in studies reporting Odds Ratios (OR) and Risk Ratios (RR)*

**All-cause**

**External**

*Effect sizes shown may vary by 0.1 to what is reported in the studies, due to logarithmic transformation during analyses

**Supplementary Appendix 9.** Results from subgroup analyses by sex, in studies reporting Hazard Ratios (HR)*

**Both**

**Men**

*Effect sizes shown may vary by 0.1 to what is reported in the studies, due to logarithmic transformation during analyses

 **Supplementary Appendix 10.** Results from subgroup analyses by follow-up period, in studies reporting Hazard Ratios (HR)*

**≤ 10 years**

**10-20 years**

**≥ 20 years**

*Effect sizes shown may vary by 0.1 to what is reported in the studies, due to logarithmic transformation during analyses

**Supplementary Appendix 11A.** Results from subgroup analyses by methodological quality, in studies reporting Odds Ratios (OR) and Risk Ratios (RR)*

**Fair**

**Good**

*Effect sizes shown may vary by 0.1 to what is reported in the studies, due to logarithmic transformation during analyses

**Supplementary Appendix 11B.** Results from subgroup analyses by methodological quality, in studies reporting Hazard Ratios (HR)*

**Fair**

**Good**

*Effect sizes shown may vary by 0.1 to what is reported in the studies, due to logarithmic transformation during analyses
